# DNA Barcoding Reveals Species Diversity and Host Associations of Dryinidae Wasps (Insecta, Hymenoptera): A Case Study from the Xisha Islands in the South China Sea

**DOI:** 10.3390/ani14243587

**Published:** 2024-12-12

**Authors:** Huayan Chen, Massimo Olmi, Jun Wang, Qilin Sun, Shixiao Luo

**Affiliations:** 1 Guangdong Provincial Key Laboratory of Applied Botany, South China Botanical Garden, Chinese Academy of Sciences, Guangzhou 510650, China; huayanc@scbg.ac.cn (H.C.); wxj@scbg.ac.cn (J.W.); sunql@scbg.ac.cn (Q.S.); 2 State Key Laboratory of Plant Diversity and Specialty Crops, South China Botanical Garden, Chinese Academy of Sciences, Guangzhou 510650, China; 3 South China National Botanical Garden, Guangzhou 510650, China; 4 Tropical Entomology Research Center, Via De Gasperi 10, 01100 Viterbo, Italy; olmi@unitus.it

**Keywords:** *Anteon*, *Gonatopus*, parasitoid–host interaction, biological control, conservation

## Abstract

Dryinidae are a cosmopolitan group of important parasitoids that attack the nymphs and adults of Auchenorrhyncha (Hemiptera) pests. Effective application of these parasitoids as biological control agents against Auchenorrhyncha and the development of conservation strategies depend upon a comprehensive understanding of the diversity and host interaction networks of these parasitoids. However, traditional morphology-based identification and rearing methods are inadequate to understand the diversity and host associations of dryinids due to their extreme sexual dimorphism, species rarity, and unique biology. To address this, we assessed the effectiveness of DNA barcode techniques for surveying dryinid diversity and their host associations on the Xisha Islands in the South China Sea. By analyzing the mitochondrial cytochrome c oxidase subunit I (*COI*) sequencies of adults and larvae of dryinids collected on six islets of the Xisha Islands, we recognized 11 dryinid species, including an undescribed species and one re-instated species, *A. malaysianum* Olmi, 1987, **stat. rev.** Additionally, we confirmed the host associations of each dryinid species and constructed a dryinid–hopper interaction network. This network was mostly composed of specialists, suggesting that the population sizes of some dryinid species were extremely small and highlighting the need for further conservation assessments of dryinids on the Xisha Islands. Overall, our study demonstrates the efficacy of DNA barcoding methods for assessing parasitoid diversity and their host associations, which are crucial for guiding conservation and management efforts for these important insects.

## Abstract (plain language summary)

Dryinidae are a diverse family of parasitoids, and they are important natural enemies of Auchenorrhyncha (Hemiptera) pests. A comprehensive understanding of the diversity and host interaction networks of these parasitoids is critical to their application in biological control programs and their conservation. However, traditional methods such as morphological identification and rearing are insufficient to understand the diversity and host associations of dryinids. In this paper, we assessed the effectiveness of DNA barcode techniques for surveying dryinid diversity and their host associations on the Xisha Islands. Based on the analyses of the mitochondrial cytochrome c oxidase subunit I (*COI*) sequences of adults and larvae, we recognized 11 dryinid species, including an undescribed species and one re-instated species, on the Xisha Islands. We also confirmed the host associations of each dryinid species and constructed a dryinid–hopper interaction network which suggests that the population sizes of some dryinid species are extremely small and further conservation assessments of dryinids for the Xisha Islands are needed. Our study demonstrates that DNA barcoding methods are potent for assessing parasitoid diversity and their host associations.

## 1. Introduction

Dryinidae is a cosmopolitan wasp family, with over 1900 species found worldwide [1,2,3]. These wasps are parasitoids and predators of Auchenorrhyncha (Hemiptera) pests, which can cause serious problems in crops such as rice, sugarcane, and fruits via direct feeding or by transmitting pathogens [4]. By parasitism and predation, these wasps are promising biological control agents that can be used to reduce the populations of leafhopper pests [5], and some species have been implemented in biological control programs [4].

Despite their importance in controlling pests, the diversity of these parasitoids and their host associations remain poorly understood, which has hampered their application in biological control and the development of conservation strategies [6,7]. Sexual dimorphism is extreme in Dryinidae, and many species were described based primarily on a single sex due to the difficulty in associating females with males [8,9,10]. This practice (i.e., describing a new species based on a single sex) would lead to an inaccurate understanding of the real diversity of these parasitoids [3]. Furthermore, the host associations of dryinid wasps are even more difficult to determine because this usually requires rearing the wasps from their hosts, which is challenging due to the difficulty of keeping the parasitized host alive until the emergence of the wasp(s) or because many dryinid wasps themselves are extremely rare, and it is rarely possible to locate and collect live parasitized hosts [7].

With the application of DNA barcoding methods, however, the aforementioned obstacle could be overcome. Studies have demonstrated that DNA barcoding is potent in species delimitation and uncovering the female–male association of the same species, especially in sexually dimorphic dryinids, which would enhance our understanding of their diversity [2,3,9,10,11]. Most dryinids are ectoparasitoids of the nymphs or adults of their host, and the larvae of the wasps insert their heads into the host’s coelom to feed on the hemolymph and tissues of the host from outside, with the rest of their body protruding between two of the host’s sclerites [4]. This specialized biology provides a rich resource for confirming the host associations of Dryinidae using DNA barcoding techniques. By analyzing the mitochondrial cytochrome c oxidase subunit I (*COI*) gene of the wasp adult and larva, the hosts of several dryinid species have been discovered [7,10]. This approach should be further tested on a broad scale to evaluate its efficacy.

Oceanic islands, due to their isolated habitat and the sensitivity of their biota, provide excellent opportunities for exploring questions related to biodiversity and conservation [12]. Previously, DNA barcoding was used to confirm the host of the first Dryinidae species (*Gonatopus viet* Olmi, 1986) on the Xisha Islands in the South China Sea [7]. During recent expeditions to the Xisha Islands, we collected a large number of Dryinidae and parasitized hopper specimens, which provided us with plenty of material to assess the diversity of dryinids and evaluate their conservational status. Thus, this study aims to investigate the diversity and host association of dryinid wasps on the Xisha Islands using DNA barcoding.

## 2. Materials and Methods

### 2.1. Insect Collection

**Figure 1:**
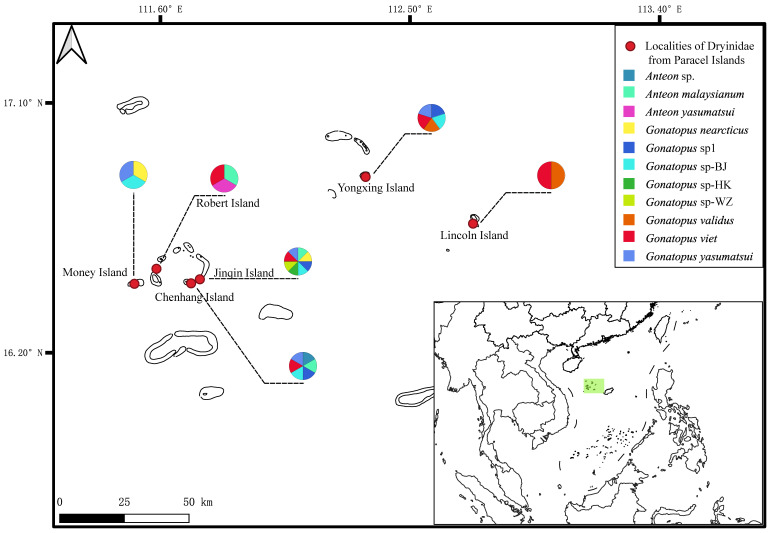
Sampling sites and distribution of Dryinidae species collected in the Xisha Islands.

This work is based upon specimens collected by sweep nets and yellow pan traps during two field trips to the Xisha Islands (Paracel Islands), which are a series of coral islets in the South China Sea. Samples of Dryinidae and their hosts were collected in various habitats (Figure S1) on six islets (Figure 1 (The sampling map was drawn using Q-GIS software (3.38.3) [13]); Table S1). The two field trips were carried out from 17 April to 12 May in 2019 and 4–16 January in 2024. Yellow pan traps (yellow plastic bowl, 4 cm height × 15 cm inside diameter, filled with soapy water, Figure S2A) were placed on the ground for 24 h to allow for the collection of insects which were preserved in 100% alcohol. A bottom-sealed emergence tent (dimensions: L 110 cm × H 110 cm × W 110 cm; built from a white fabric with an opening attached to a collecting bottle on the top and a dome-shaped zipper on the side for access, Figure S2B) was used to collect the specimens obtained with a sweep net. A sweep net was used to collect random insects on vegetation, and the material collected was placed into the emergence tent to allow most of the insects to become trapped in a collecting bottle filled with 100% alcohol. This method is efficient in collecting a large amount of insect specimens and can cover a large sampling area in a short time period. The specimens collected by both sampling methods were then used for DNA sequencing and morphological examinations.

### 2.2. Species Identification

Adult dryinid wasps and parasitized hosts (larva or adult) were first sorted into morphospecies. Representative specimens of each wasp and host species (Table 1) were selected for DNA sequencing and morphological examination. The wasp larva of each parasitized host specimen (Table 2) was also used for DNA analysis to confirm its identity. Each parasitized host specimen was photographed to record the position of the attached wasp larva (called a thylacium) using a Nikon SMZ25 microscope with a Nikon DS-Ri 2 digital camera system (Nikon Corporation, Tokyo, Japan) before DNA extraction. Genomic DNA of the adult wasps, their hosts (larva or adult), and wasp larva was extracted from the whole body, a single hind leg, and the cuticle, respectively, using a TIANamp Micro DNA Kit (Tiangen Biotech, Beijing, China) as described previously [3,7]. The sequences of the *COI* gene were acquired as described in Chen et al. [3]. All sequences generated in this study were deposited in GenBank under accession numbers listed in Table 1 and Table 2.

Voucher specimens (Table 1) were deposited in the insect collection of South China Botanical Garden, Chinese Academy of Sciences, Guangzhou, China (SCBG). Species of adult dryinid wasps were first identified based on morphological characteristics using the available keys present in Xu et al. [14] and Olmi and Xu [15], and then the genetic distance among species based on the *COI* sequences were analyzed to confirm their identities. Sequences were aligned by codons using MUSCLE implemented in Geneious 11.0.3, and the K2P distances within and between species were calculated.

Species descriptions followed the terminology used by Olmi et al. [1]. Body length was measured in millimeters from the anterior-most portion of the head (excluding the antenna) to the tip of the metasoma (excluding the sting, if exserted). Other measurements presented are relative. The abbreviations used in the text are as follows. POL: distance between inner edges of the lateral ocelli; OL: distance between inner edges of a lateral ocellus and the median ocellus; OOL: distance from the outer edge of a lateral ocellus to the eye; OPL: distance from the posterior edge of a lateral ocellus to the occipital carina; TL: distance from the posterior edge of an eye to the occipital carina.

Multifocal images of voucher specimens were produced using a Nikon SMZ25 microscope with a Nikon DS-Ri 2 digital camera system (Melville, NY, USA). The female chela and male genitalia were dissected using insect pins and gold–palladium coated for examination in a JSM-6360LV scanning electron microscope (JEOL, Tokyo, Japan). The chela and genitalia were then mounted on the point together with the original specimen. Images were then post-processed with Adobe Photoshop CS6 Extended (Adobe, San Jose, CA, USA).

Species of adult hosts were identified to the lowest possible taxonomic level by leafhopper and planthopper taxonomists (Qingquan Xue and Yixin Huang, see Acknowledgements), while the identity of nymphal hosts was confirmed by comparison of *COI* sequences. DNA sequences of wasp larvae were blasted against the GenBank database to check for identification and were also compared with a reference *COI* data set of Chinese Dryinidae (including the sequences of adult wasps newly generated in this study) compiled by the first author. Sequences with similarity greater than or equal to 97% were considered as the same species, because intraspecific pairwise distances of *COI* sequences in Dryinidae are generally less than 3% [2,3]. To display affinities among the studied dryinid wasps (adults and larvae), sequences were aligned by codon using MUSCLE and analyzed using RAxML as implemented in Geneious 11.0.3 to generate a maximum likelihood (ML) tree. The GTRGAMMA +I model was used as the best nucleotide substitution model, which was determined with ModelFinder [16] with the merge option enabled. Automatic bootstrapping criterion was selected as the appropriate number of bootstraps; 1000 replicates were run. The *COI* sequence of *Chrysis principalis* Smith, 1874 (Hymenoptera, Chrysididae) (GenBank: KY430781) was used as an outgroup based on the phylogenetic topologies recovered by Tribull [17].

### 2.3. Parasitoid–Host Networks

We used the package bipartite [18] to plot the parasitoid–host interaction network and calculate the network-level indices in R4.4.1 [19]. Three network-level indices were calculated to illustrate the structural properties of the network [20]. Connectance is the fraction of all possible links that are realized in a network, and it increasies with network generalization [21]. Network-level specialization (H’2) describes the degree of specialization or partitioning among two parties in the entire network [22], ranging from 0 (low specialization) to 1 (high specialization). Nestedness quantifies the degree of interactions of specialized species in a network.

## 3. Results

### 3.1. Wasp Adult Identification

The thirteen newly generated *COI* sequences of the adult wasps ranged from 675 bp to 681 bp. When analyzed in the BOLD system [23] and GenBank database, the blast results of the sequences generated in this study returned no matching sequences, with similarities that were all less than 93.5%. The intraspecific pairwise distances ranged from 0 to 1%, while the interspecific distances ranged between 7.5% and 20.4% (Table S2). Thus, based on both morphology and *COI* sequences, the adult wasp specimens collected on the six sampled islets were identified into seven morphospecies, including one probably undescribed species (description provided below), two known species of the genus *Anteon* Jurine, three known species, and an unidentified species (only male specimens, which are not identifiable morphologically) of the genus *Gonatopus* Ljungh (Table 2; Figures S3–S10). *Anteon malaysianum* Olmi, 1987 was first described from Malaysia but later was treated as a synonym of *Anteon yasumatsui* Olmi, 1984, due subtle differences in their mesoscutum but almost identical chela of the female foreleg [24]. In this study, the *COI* sequences, along with morphological characteristics such as the color of the antennae and legs and the sculpture of the mesosoma, indicate that *A. malaysianum* is a different species from *A. yasumatsui.* Therefore, we treat *A. malaysianum* Olmi, 1987, **stat. rev.**, as a valid species. With the help of DNA barcoding, the male of *Gonatopus validus* (Olmi, 1984) was first discovered.

We here provide a description of the specimen to facilitate its identification in the future.

*Anteon* sp.

Material examined. CHINA: Hainan, Shansha, Chenhang Island, 16°26′59.982″ N, 111°42′39.054″ E, 10.i.2024, sweep, Huayan Chen leg., DNA: PP600740, SCBG_E0009677 (SCBG).

**Figure 2:**
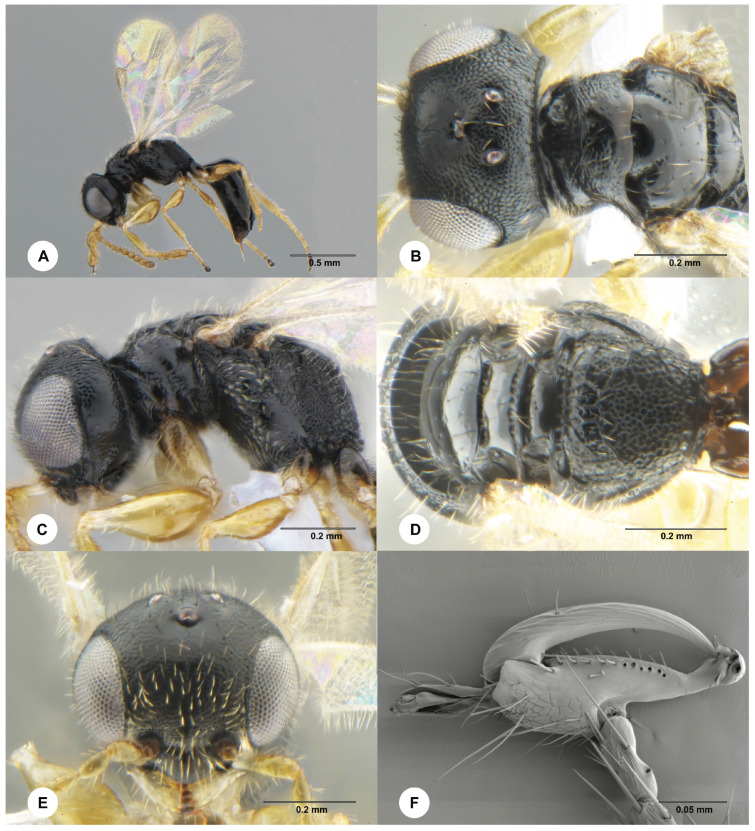
*Anteon* sp., female (SCBG_E0009677). (**A**) Habitus, lateral view. (**B**) Head and anterior mesosoma, dorsal view. (**C**) Mesosoma, lateral view. (**D**) Posterior mesosoma, dorsal view. (**E**) Head, frontal view. (**F**) Chela.

Description. Female, holotype. Body length 2.8 mm; fully winged (Figure 2A). Head black with mandible light yellow; antenna yellow; mesosoma and metasoma black; legs yellow with proximal extremity of coxae dark brown to black. Antenna clavate; antennomeres in the following proportions: 11:6:4:3.5:3.5:3.5:4:4:5:8. Head (Figure 2B,C) dull, largely granulate with smooth areas below eyes; vertex without two oblique keels connecting lateral ocelli to occipital carina; frontal line absent; OL = 6; OOL = 8; OPL = 6; POL = 9; TL = 8; greatest breadth of lateral ocellus shorter than OPL (1:3); occipital carina complete. Pronotum largely granulate, with the anterior surface about as long as the dorsal surface; dorsal surface shiny posteriorly, much shorter than mesoscutum (9:14); pronotal tubercle reaching tegula. Mesoscutum and mesoscutellum shiny, largely smooth. Notauli incomplete, present at the anterior 0.5 of the mesoscutum, foveate (Figure 2B). Metanotum shiny, smooth. Metapectal–propodeal complex with strong transverse keel between disc and propodeal declivity; disc reticulate rugose; propodeal declivity reticulate rugose, without longitudinal keels (Figure 2D). Forewing hyaline; distal part (Rs) of the stigmal vein (2r-rs&Rs) shorter than the proximal (2r-rs) part (3:5). Protarsomeres in the following proportions: 6:2:4:5:13. Protarsomere 2 produced into a hook. Enlarged claw (Figure 2F) with proximal prominence bearing one long bristle. Protarsomere 5 (Figure 2F) with basal part shorter than distal part, with one row of 11 lamellae, in addition to scattered hairs and bristles; distal apex with four lamellae. Tibial spurs 1/1/2.

### 3.2. Wasp Larva Identification

**Figure 3:**
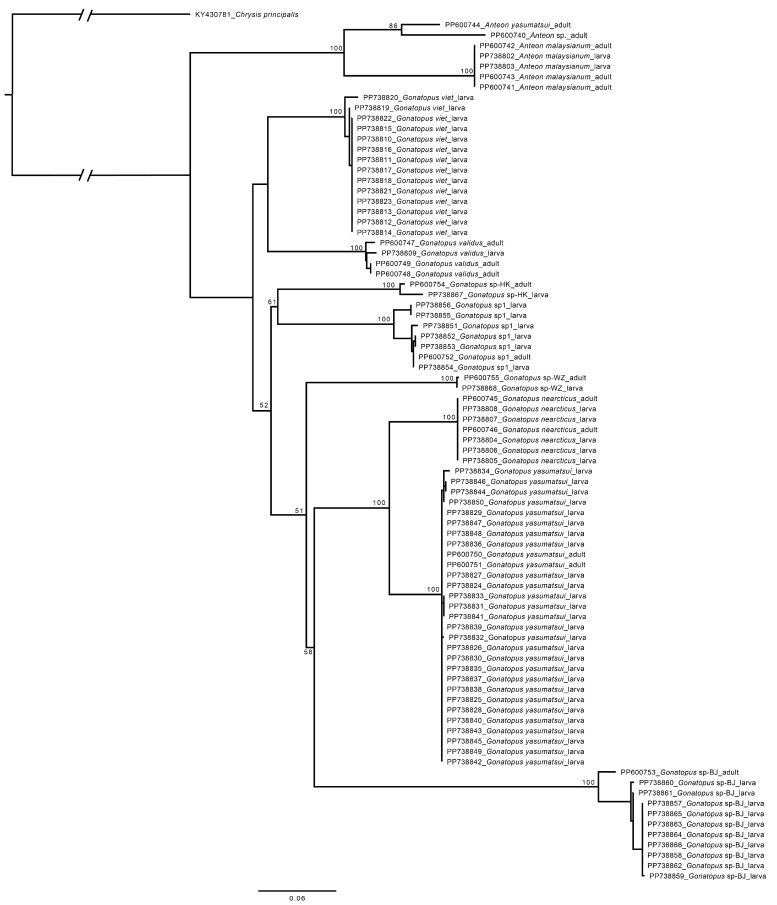
Maximum Likelihood tree showing relationships between the studied dryinid species based on codon-aligned nucleotide sequences of *COI*. Bootstrap values of 50 and above are indicated at nodes. The scale bar represents 0.06 substitutions per site.

In total, 67 parasitized hosts representing two families and nine species were subjected to a molecular analysis of the wasp larva. Each host specimen bore a single wasp larva. Based on the comparison of the *COI* sequences of each wasp larva with a reference *COI* data set of Chinese Dryinidae and a species delimitation threshold of 97% similarity, the 67 wasp larvae were sorted into nine species, including one known species of *Anteon* and four known and four unidentifiable (male only) species of *Gonatopus* (Table 2). The affinities among the larvae and adults of the studied species are shown in Figure 3. Of the four unidentifiable species of *Gonatopus*, the *COI* sequences of three species were matched with male specimens collected outside of the Xisha Islands, indicating a more comprehensive reference *COI* data set of adult wasps would facilitate the identification of the wasp larvae.

**Figure 4:**
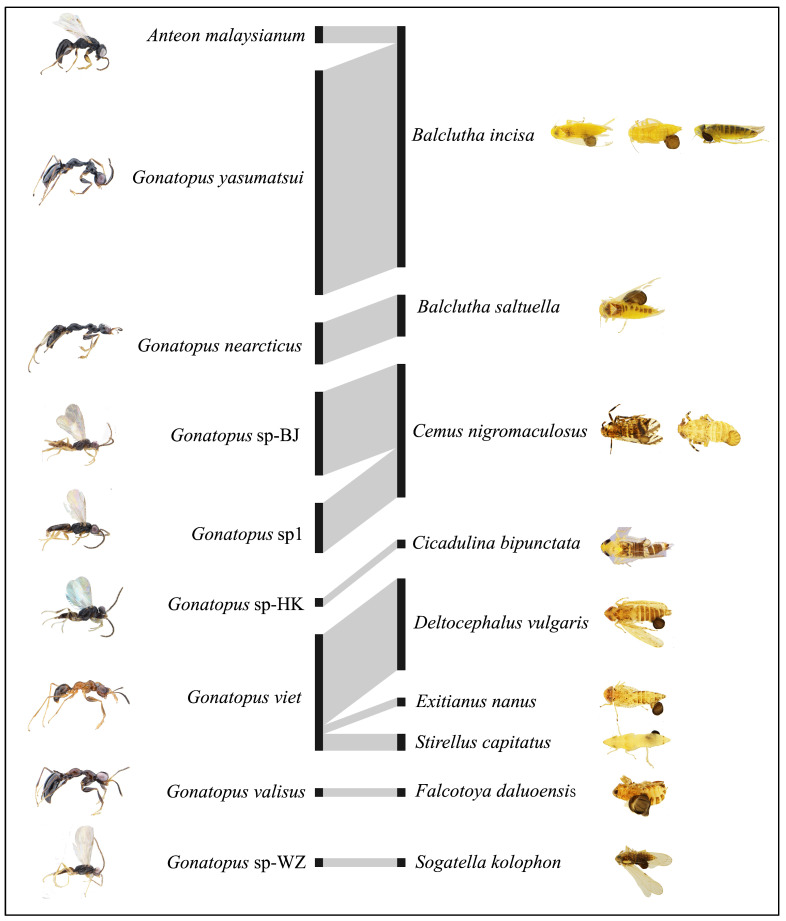
Bipartite network representations of Dryinidae (**left column**) andhopper (**right column**) associations on the Xisha Islands. The rectangles represent dryinid species (**left column**) and host species (**right column**), and the connecting lines represent interactions between wasp and host species. The width of rectangles is proportional to species abundance, while the width of lines indicates interaction frequency.

*Anteon malaysianum* was found to parasitize the female adult of *Balclutha incisa* (Kirschbaum), with the thylacium positioned between the pro- and mesothoraxes. Four species of *Gonatopus* (*G. nearcticus*, *G. validus*, *Gonatopus* sp-HK, and *Gonatopus* sp-WZ) were found parasitizing the adult of their host(s), whereas the other four species (*G. viet*, *G. yasumatsui*, *Gonatopus* sp1, and *Gonatopus* sp-BJ) were found parasitizing both the adults and nymphs. The thylacium position of the eight *Gonatopus* species is on the dorsal or ventral metasoma (Table 2; Figure 4).

### 3.3. Parasitoid–Host Interactions

Parasitoid–host interactions are presented in the bipartite network of species that occur on the Xisha Islands. A total of 67 links were established based on nine dryinid species and nine hopper species (Figure 4; Table 2). The majority of the dryinid species (78%) parasitize one single hopper species, with the exception of *Gonatopus viet*, which parasitizes three species. *Gonatopus yasumatsui* was the species with the highest occurrence (41%) of all interactions, followed by *G*. *viet* and *Gonatopus* sp-BJ. Only two hopper species, *B*. *incisa* and *Cemus nigromaculosus* (Muir, 1917), were parasitized by two different wasp species, while the remaining seven hopper species were each parasitized by only one wasp species each. Among the nine hopper species, *B. incisa* was the most susceptible to parasitism. Overall, the network exhibited a low connectance (0.14), a low nestedness (28.47), and a high specialization (0.98) (Table S4).

## 4. Discussion

In this study, we discovered and identified eleven species of dryinid wasps from the Xisha Islands. This identification was achieved through a comprehensive analysis that incorporated both adult wasp morphology and DNA sequences from both adults and larvae. Specifically, seven species were identified based on adult wasps, while nine species were identified from the larvae. The application of DNA barcoding significantly enhanced our understanding of the diversity of dryinid species inhabiting the Xisha Islands, particularly in the genera *Anteon* and *Gonatopus*, which are two of the most speciose genera of Dryinidae.

For the genus *Anteon*, the validity of *A. malaysianum* was reinstated and an undescribed species was discovered. The undescribed *Anteon* species is morphologically similar to *A. yasumatsui* in body size and chela structure, but it is different from *A. yasumatsui* in having antennomeres four to seven times broader than long and coxae that are dark brown to black. The intraspecific pairwise distances between this undescribed species and *A. yasumatsui* is 7.5%, also suggesting that it is a distinctive species different from *A. yasumatsui* and probably representing a new species. However, we only have one specimen of this undescribed species, and more material is needed to confirm its identity. Traditionally, the morphological taxonomy of *Anteon* depends heavily on the chela of the female foreleg [1,13,14]. However, the validity of a species defined by the traditionally used characteristics, such as the length of notaulus and the structure of female chela, should be tested by rearing adult wasps from their host(s) or DNA analysis [2]. Among the three *Anteon* species found in this study, the female chelae are morphologically uniform, but their identities were confirmed by the combined analyses of the *COI* sequences and other morphological characteristics such as the color of the antennae and legs and the sculpture of the mesosoma. This indicates that DNA barcoding can aid in identifying valuable diagnostic morphological characteristics, thereby enhancing morphological species delimitation. DNA barcoding also serves as the bridge that facilitates the recognition of the members of the opposite sex [2,3,9,10,11]. For example, sexual dimorphism is particularly extreme in the genus *Gonatopus*: females of most species are wingless and males are fully winged. The adult males alone of many *Gonatopus* species are not identifiable, and most species have been described based solely on their females [1]. The sexual association of many *Gonatopus* species has previously been confirmed by rearing, although a few species have been confirmed with DNA sequences [9]. In this study, we confirmed the female–male association of two *Gonatopus* species, *G. nearcticus* and *G. validus*. In addition, the male of *G. validus* was discovered for the first time, indicating that DNA barcoding is a powerful tool to enhance the sexual association of the same species.

In total, 67 wasp larvae had their DNA barcodes sequenced and analyzed, leading to the identification of nine dryinid wasp species. However, only five of these species could be definitively assigned to a specific species. A more comprehensive reference *COI* data set of adult dryinid wasps in the future will help to confirm the host association of the four unidentifiable species of *Gonatopus* identified in this study [7]. Additional morphological characteristics of the developing wasp larvae within the host can also be used in genus and species determination. The position of the thylacium is generally characteristic of a dryinid taxa [25], but it can vary depending on the species and size of the host(s) [4]. Our findings are congruent with this perspective. The leafhoppers parasitized by *Anteon* species usually have the thylacium situated between the prothorax and mesothorax [1], which was the placement observed for *A. malaysianum*, whereas the thylacium position of the eight *Gonatopus* species was variable but always on the metasomal segments, positioned ventrally or dorsally.

The accurate identification of the host is just as important as the accurate identification of the parasitoid and allows for the categorization of the parasitoid as a generalist (parasitizes many host species) or specialist (parasitizes a single host species, genus, or family). The host of *A. malaysianum* was previously unrecorded, as it was treated as a synonym of *A. yasumatsui*, which has been reported to parasitize multiple host species [6]. In this study, we confirmed that *B. incisa* is the host of *A*. *malaysianum*. The host (*F. daluoensis*) of *G. validus* was discovered for the first time in this study. Additionally, using DNA barcoding, the host of *G. viet* was initially identified as *Stirellus capitatus* [7], but two additional host species, *Deltocephalus vulgaris* Dash & Viraktamath, 1998 and *Exitianus nanus* Distant, 1908, were discovered in this study, indicating that *G. viet* is a generalist. Although *G. nearcticus* and *G. yasumatsui* were recorded to parasitize *B. saltuella* and *B. incisa*, respectively, in this study, they previously have been reported as generalists [6]. Previous studies suggest that many dryinid species can parasitize different groups of hosts, and the current records of monophagous parasitism may be due to the insufficient investigation of parasitoid–host associations [7,26,27]. At least one study also suggested that unstable ecological conditions could lead to dryinid wasps colonizing accidental hosts [28]. Considering the small islet areas (Table 1) and limited host species available on the Xisha Islands, we would expect a generalist bias in the dryinid community, but we found more specialists (most species only parasitize one single host species) than generalists. This pattern may be due to our limited sample sizes, as some parasitoid–host interactions were found based on one or a few specimens. Nevertheless, our findings at least indicate that the population sizes of some dryinid species are extremely small on the Xisha Islands.

Island species usually have a small population size, and thus are more vulnerable to extinction [12], especially for species at high trophic levels. The dryinid–hopper interaction network of the Xisha Islands had a low connectance and nestedness, but a high specialization, suggesting that the network is unstable and some specialists are at risk of extinction. However, this conclusion should be viewed with some caution because the metrics measured from such interaction networks are sensitive to sampling bias, and our sampling effort was limited. The climate of the Xisha Islands can be categorized into two seasons, the dry season (October to March) and the rainy season (April to September), and studies have found that at least plant–pollinator networks show seasonal variations [29], which is most likely true for the parasitoid–host networks, as well. Nevertheless, the methods we used in this study provide an opportunity to recognize the interacting relationships of species at high trophic levels, which may help to develop better conservation strategies. Dryinids are the natural enemies of hoppers, which are pests of various plants on the islands; therefore, dryinids are important biological control agents to keep hopper pests in check [5]. One of the strategies to make use of dryinids in pest biological control is to develop conservation plans for local species. An accurate species identification and the confirmation of host associations are essential for developing conservation strategies.

## 5. Conclusions

By using an integrated taxonomic approach that combines morphological identification with DNA barcoding methods, we recognized 11 dryinid species present on the Xisha Islands, including a new species of *Anteon*. Furthermore, the hosts of most of the dryinid species and the characteristics of their interaction network were identified via DNA sequence analyses. Our study demonstrates the effectiveness of DNA barcoding methods in uncovering parasitoid diversity and assessing their host associations, which are crucial for devising conservation management strategies, particularly for taxa inhabiting oceanic islands.

## Figures and Tables

**Table 1:** List of sequenced adult parasitoids and hosts.

Code	Family	Species	Sex	Locality	GenBank Accession No.
		Parasitoid			
SCBG_E0009677	Dryinidae	*Anteon* sp.	female	Chenhang Is.	PP600740
SCBG_E0009673	Dryinidae	*Anteon malaysianum* Olmi, 1987	female	Jinqin Is.	PP600741
SCBG_E0009676	Dryinidae	*Anteon malaysianum* Olmi, 1987	female	Chenhang Is.	PP600742
SCBG_E0009679	Dryinidae	*Anteon malaysianum* Olmi, 1987	female	Ganquan Is.	PP600743
SCBG_E0009680	Dryinidae	*Anteon yasumatsui* Olmi, 1984	female	Ganquan Is.	PP600744
SCBG_E0009681	Dryinidae	*Gonatopus nearcticus* (Fenton, 1905)	female	Jinyin Is.	PP600745
SCBG_E0009682	Dryinidae	*Gonatopus nearcticus* (Fenton, 1905)	male	Jinyin Is.	PP600746
SCBG_E0009671	Dryinidae	*Gonatopus validus* (Olmi, 1984)	male	Yongxing Is.	PP600747
SCBG_E0009674	Dryinidae	*Gonatopus validus* (Olmi, 1984)	female	Lincoln Is.	PP600748
SCBG_E0009675	Dryinidae	*Gonatopus validus* (Olmi, 1984)	male	Lincoln Is.	PP600749
SCBG_E0009672	Dryinidae	*Gonatopus yasumatsui* Olmi 1984	female	Jinqin Is.	PP600750
SCBG_E0009678	Dryinidae	*Gonatopus yasumatsui* Olmi 1984	female	Chenhang Is.	PP600751
SCBG_E0009670	Dryinidae	*Gonatopus* sp1	male	Yongxing Is.	PP600752
SCAU_3011792	Dryinidae	*Gonatopus* sp-BJ	male	Beijing	PP600753
SCAU_3048892	Dryinidae	*Gonatopus* sp-HK	male	Haikou	PP600754
SCAU_3044110	Dryinidae	*Gonatopus* sp-WZ	male	Wenzhou	PP600755
		Host			
SCBG_E0009800	Cicadellidae	*Balclutha incisa* (Matsumura, 1902)	female	Jinqin Is.	PP600756
SCBG_E0009803	Cicadellidae	*Balclutha incisa* (Matsumura, 1902)	female	Jinqin Is.	PP600757
SCBG_E0009808	Cicadellidae	*Balclutha incisa* (Matsumura, 1902)	male	Jinqin Is.	PP600758
SCBG_E0009824	Cicadellidae	*Balclutha incisa* (Matsumura, 1902)	male	Chenhang Is.	PP600759
SCBG_E0009827	Cicadellidae	*Balclutha saltuella* (Kirschbaum, 1868)	female	Jinyin Is.	PP600760
SCBG_E0009828	Cicadellidae	*Balclutha saltuella* (Kirschbaum, 1868)	male	Jinyin Is.	PP600761
SCBG_E0009950	Cicadellidae	*Cicadulina bipunctata* (Melichar 1904)	male	Jinqin Is.	PP600762
SCBG_E0009777	Cicadellidae	*Deltocephalus vulgaris* Dash & Viraktamath, 1998	male	Yongxing Is.	PP600763
SCBG_E0009811	Cicadellidae	*Exitianus nanus* (Distant, 1908)	male	Lincoln Is.	PP600764
SCBG_E0009955	Cicadellidae	*Exitianus nanus* (Distant, 1908)	female	Lincoln Is.	PP600765
SCBG_E0009825	Cicadellidae	*Stirellus capitatus* (Distant, 1918)	male	Yongxing Is.	PP600766
SCBG_E0009781	Delphacidae	*Cemus nigromaculosus* (Muir, 1917)	male	Yongxing Is.	PP600767
SCBG_E0009791	Delphacidae	*Cemus nigromaculosus* (Muir, 1917)	male	Yongxing Is.	PP600768
SCBG_E0009832	Delphacidae	*Falcotoya daluoensis* Ding, 2006	female	Lincoln Is.	PP600769
SCBG_E0009833	Delphacidae	*Sogatella kolophon* (Kirkaldy, 1907)	female	Jinqin Is.	PP600770

Note: Is. = Island.

**Table 2:** List of sequenced parasitoid nymphs.

Code	Parasitoid Species	Host Species	Host Instar/Sex	Thylacium Position	Locality	GenBank Accession No.
SCBG-E0009800	*A. malaysianum*	*B. incisa*	adult/female	between pro- and mesothorax	Jinqin Is.	PP738802
SCBG-E0009895	*A. malaysianum*	*B. incisa*	adult/female	between pro- and mesothorax	Chenhang Is.	PP738803
SCBG-E0009827	*G. nearcticus*	*B. saltuella*	adult/female	between T2 and T3	Jinyin Is.	PP738804
SCBG-E0009828	*G. nearcticus*	*B. saltuella*	adult/male	between T2 and T3	Jinyin Is.	PP738805
SCBG-E0009834	*G. nearcticus*	*B. saltuella*	adult/female	between T1 and T2	Jinqin Is.	PP738806
SCBG-E0009946	*G. nearcticus*	*B. saltuella*	adult/female	between T2 and T3	Jinqin Is.	PP738807
SCBG-E0009947	*G. nearcticus*	*B. saltuella*	adult/male	between T3 and T4	Jinqin Is.	PP738808
SCBG-E0009832	*G. validus*	*F. daluoensis*	adult/female	between S2 and S3	Lincoln Is.	PP738809
SCBG-E0009777	*G. viet*	*D. vulgaris*	adult/male	between S4 and S5	Yongxing Is.	PP738810
SCBG-E0009778	*G. viet*	*D. vulgaris*	adult/male	between S4 and S5	Yongxing Is.	PP738811
SCBG-E0009942	*G. viet*	*D. vulgaris*	adult/male	between S3 and S4	Yongxing Is.	PP738812
SCBG-E0009943	*G. viet*	*D. vulgaris*	adult/male	between S4 and S5	Yongxing Is.	PP738813
SCBG-E0009944	*G. viet*	*D. vulgaris*	adult/male	between S4 and S5	Yongxing Is.	PP738814
SCBG-E0009779	*G. viet*	*D. vulgaris*	adult/male	between S5 and S6	Lincoln Is.	PP738815
SCBG-E0009780	*G. viet*	*D. vulgaris*	adult/male	between S4 and S5	Lincoln Is.	PP738816
SCBG-E0009787	*G. viet*	*D. vulgaris*	adult/male	between S4 and S5	Ganquan Is.	PP738817
SCBG-E0009789	*G. viet*	*D. vulgaris*	adult/male	between S4 and S5	Ganquan Is.	PP738818
SCBG-E0009788	*G. viet*	*D. vulgaris*	adult/male	between S4 and S5	Chenhang Is.	PP738819
SCBG-E0009948	*G. viet*	*D. vulgaris*	adult/male	between S3 and S4	Jinqin Is.	PP738820
SCBG-E0009811	*G. viet*	*E. nanus*	nymph/male	between S4 and S5	Lincoln Is.	PP738821
SCBG-E0009825	*G. viet*	*S. capitatus*	nymph/female	between S3 and S4	Yongxing Is.	PP738822
SCBG-E0009945	*G. viet*	*S. capitatus*	nymph/female	between S5 and S6	Lincoln Is.	PP738823
SCBG-E0009797	*G. yasumatsui*	*B. incisa*	adult/female	between T2 and T3	Jinqin Is.	PP738824
SCBG-E0009798	*G. yasumatsui*	*B. incisa*	adult/female	between T3 and T4	Jinqin Is.	PP738825
SCBG-E0009799	*G. yasumatsui*	*B. incisa*	adult/female	between T3 and T4	Jinqin Is.	PP738826
SCBG-E0009801	*G. yasumatsui*	*B. incisa*	adult/male	between T3 and T4	Jinqin Is.	PP738827
SCBG-E0009802	*G. yasumatsui*	*B. incisa*	nymph/female	between S4 and S5	Jinqin Is.	PP738828
SCBG-E0009803	*G. yasumatsui*	*B. incisa*	nymph/female	between S4 and S5	Jinqin Is.	PP738829
SCBG-E0009804	*G. yasumatsui*	*B. incisa*	nymph/male	between S4 and S5	Jinqin Is.	PP738830
SCBG-E0009805	*G. yasumatsui*	*B. incisa*	nymph/female	between S4 and S5	Jinqin Is.	PP738831
SCBG-E0009806	*G. yasumatsui*	*B. incisa*	nymph/male	between S3 and S4	Jinqin Is.	PP738832
SCBG-E0009807	*G. yasumatsui*	*B. incisa*	nymph/male	between S4 and S5	Jinqin Is.	PP738833
SCBG-E0009808	*G. yasumatsui*	*B. incisa*	nymph/male	between S5 and S6	Jinqin Is.	PP738834
SCBG-E0009809	*G. yasumatsui*	*B. incisa*	nymph/male	between S3 and S4	Jinqin Is.	PP738835
SCBG-E0009810	*G. yasumatsui*	*B. incisa*	nymph/female	between S4 and S5	Jinqin Is.	PP738836
SCBG-E0009812	*G. yasumatsui*	*B. incisa*	adult/male	between T2 and T3	Chenhang Is.	PP738837
SCBG-E0009813	*G. yasumatsui*	*B. incisa*	nymph/female	between S3 and S4	Chenhang Is.	PP738838
SCBG-E0009814	*G. yasumatsui*	*B. incisa*	nymph/female	between T7 and T8	Chenhang Is.	PP738839
SCBG-E0009815	*G. yasumatsui*	*B. incisa*	nymph/female	between S3 and S4	Chenhang Is.	PP738840
SCBG-E0009816	*G. yasumatsui*	*B. incisa*	nymph/female	between S5 and S6	Chenhang Is.	PP738841
SCBG-E0009817	*G. yasumatsui*	*B. incisa*	nymph/female	between S4 and S5	Chenhang Is.	PP738842
SCBG-E0009818	*G. yasumatsui*	*B. incisa*	nymph/female	between S3 and S4	Chenhang Is.	PP738843
SCBG-E0009819	*G. yasumatsui*	*B. incisa*	nymph/female	between S2 and S3	Chenhang Is.	PP738844
SCBG-E0009820	*G. yasumatsui*	*B. incisa*	nymph/female	between S3 and S4	Chenhang Is.	PP738845
SCBG-E0009821	*G. yasumatsui*	*B. incisa*	nymph/female	between S4 and S5	Chenhang Is.	PP738846
SCBG-E0009823	*G. yasumatsui*	*B. incisa*	nymph/male	between T4 and T5	Chenhang Is.	PP738847
SCBG-E0009824	*G. yasumatsui*	*B. incisa*	nymph/male	between T3 and T4	Chenhang Is.	PP738848
SCBG-E0009826	*G. yasumatsui*	*B. incisa*	adult/female	between T2 and T3	Jinyin Is.	PP738849
SCBG-E0009896	*G. yasumatsui*	*B. incisa*	adult/female	between T4 and T5	Yongxing Is.	PP738850
SCBG-E0009781	*Gonatopus* sp1	*C. nigromaculosus*	adult/male	between T3 and T4	Jinqin Is.	PP738851
SCBG-E0009782	*Gonatopus* sp1	*C. nigromaculosus*	adult/female	between T3 and T4	Chenhang Is.	PP738852
SCBG-E0009792	*Gonatopus* sp1	*C. nigromaculosus*	adult/female	between T1 and T2	Yongxing Is.	PP738853
SCBG-E0009793	*Gonatopus* sp1	*C. nigromaculosus*	nymph/male	between T2 and T3	Yongxing Is.	PP738854
SCBG-E0009829	*Gonatopus* sp1	*C. nigromaculosus*	adult/male	between S3 and S4	Yongxing Is.	PP738855
SCBG-E0009830	*Gonatopus* sp1	*C. nigromaculosus*	adult/male	between S3 and S4	Yongxing Is.	PP738856
SCBG-E0009783	*Gonatopus* sp-BJ	*C. nigromaculosus*	adult/male	between T3 and T4	Jinyin Is.	PP738857
SCBG-E0009784	*Gonatopus* sp-BJ	*C. nigromaculosus*	nymph/male	between T3 and T4	Jinyin Is.	PP738858
SCBG-E0009786	*Gonatopus* sp-BJ	*C. nigromaculosus*	nymph/male	between T3 and T4	Chenhang Is.	PP738859
SCBG-E0009822	*Gonatopus* sp-BJ	*C. nigromaculosus*	nymph/male	between T4 and T5	Chenhang Is.	PP738860
SCBG-E0009790	*Gonatopus* sp-BJ	*C. nigromaculosus*	adult/male	between T4 and T5	Yongxing Is.	PP738861
SCBG-E0009791	*Gonatopus* sp-BJ	*C. nigromaculosus*	adult/male	between T2 and T3	Yongxing Is.	PP738862
SCBG-E0009795	*Gonatopus* sp-BJ	*C. nigromaculosus*	nymph/male	between T4 and T5	Yongxing Is.	PP738863
SCBG-E0009796	*Gonatopus* sp-BJ	*C. nigromaculosus*	nymph/male	between T3 and T4	Yongxing Is.	PP738864
SCBG-E0009831	*Gonatopus* sp-BJ	*C. nigromaculosus*	nymph/male	between T3 and T4	Yongxing Is.	PP738865
SCBG-E0009949	*Gonatopus* sp-BJ	*C. nigromaculosus*	nymph/male	between T4 and T5	Jinqin Is.	PP738866
SCBG-E0009950	*Gonatopus* sp-HK	*C. bipunctata*	adult/male	between T4 and T5	Jinqin Is.	PP738867
SCBG-E0009833	*Gonatopus* sp-WZ	*S. kolophon*	adult/female	between T2 and T3	Jinqin Is.	PP738868

Note: S = sternite, T = tergite, Is. = Island.

## Data Availability

All data are available in this paper.

## Supplementary Materials

The following supporting information can be downloaded at: https://www.mdpi.com/article/10.3390/ani14243587/s1, Figure S1: Different habitats of the Xisha Islands. (A) Forest. (B) Mixed forest, shrub and herbaceous community. (C) Costal shrub and herbaceous community. (D) Herbaceous community; Figure S2: (A) Yellow pan trap. (B) Bottom-sealed emergence tent used with a sweep net and collecting tube. Figure S3. *Anteon malaysianum* Olmi, 1987, female (SCBG_E0009679). (A) Habitus, lateral view. (B) Head and mesosoma, dorsal view. (C) Head and mesosoma, lateral view. (D) Wings. (E) Head, frontal view. (F) Chela; Figure S4. *Anteon yasumatsui* Olmi, 1984, female (SCBG_E0009680). (A) Habitus, lateral view. (B) Head and mesosoma, dorsal view. (C) Head and mesosoma, lateral view. (D) Wings. (E) Head, frontal view. (F) Chela; Figur S5. *Gonatopus nearcticus* (Fenton, 1905), female (SCBG_E0009681). (A) Habitus, dorsal view. (B) Habitus, lateral view. (C) Mesosoma, dorsal view. (D) Mesosoma, lateral view. (E) Head, frontal view. (F) Chela; Figure S6. *Gonatopus nearcticus* (Fenton, 1905), male (SCBG_E0009682). (A) Habitus, larteral view. (B) Head and mesosoma, dorsal view. (C) Head and mesosoma, lateral view. (D) Wings. (E) Head, frontal view. (F) Antenna; Figure S7. *Gonatopus validus* (Olmi, 1984), female (SCBG_E0009674). (A) Habitus, dorsal view. (B) Habitus, lateral view. (C) Head and mesosoma, dorsal view. (D) Head and mesosoma, lateral view. (E) Head, frontal view. (F) Chela; Figure S8. *Gonatopus validus* (Olmi, 1984), male (SCBG_E0009671). (A) Habitus, lateral view. (B) Head and mesosoma, dorsal view. (C) Head and mesosoma, lateral view. (D) Wings. (E) Antenna. (F) Genitalia, ventral view; Figure S9. *Gonatopus yasumatsui* Olmi 1984, female (SCBG_E0009678). (A) Habitus, dorsal view. (B) Habitus, lateral view. (C) Head and mesosoma, dorsal view. (D) Head and mesosoma, lateral view. (E) Head, frontal view. (F) Chela; Figure S10. *Gonatopus* sp1, male (SCBG_E0009670). (A) Habitus, larteral view. (B) Head and mesosoma, dorsal view. (C) Head and mesosoma, lateral view. (D) Wings. (E) Head, frontal view. (F) Antenna; Table S1. Geographic coordinates and area of sampled islets; Table S2. Intraspecific pairwise distance of Dryinidae adults based on COI sequences (%); Table S3. Interspecific pairwise distance of Dryinidae adults based on COI sequences (%); Table S4. Network metrics of the parasitoid-host network on the Xisha Islands.
