# Supplementary material for: DNA Barcoding Reveals Species Diversity and Host Associations of Dryinidae Wasps (Insecta, Hymenoptera): A Case Study from the Xisha Islands in the South China Sea
Source: Animals (Basel). 2024 Dec 12;14(24):3587. doi: 10.3390/ani14243587 (PMC11672658; doi:10.3390/ani14243587)
Supplement: Supplementary file 1 [file animals-14-03587-s001.zip › animals-3280884-supplementary.pdf]

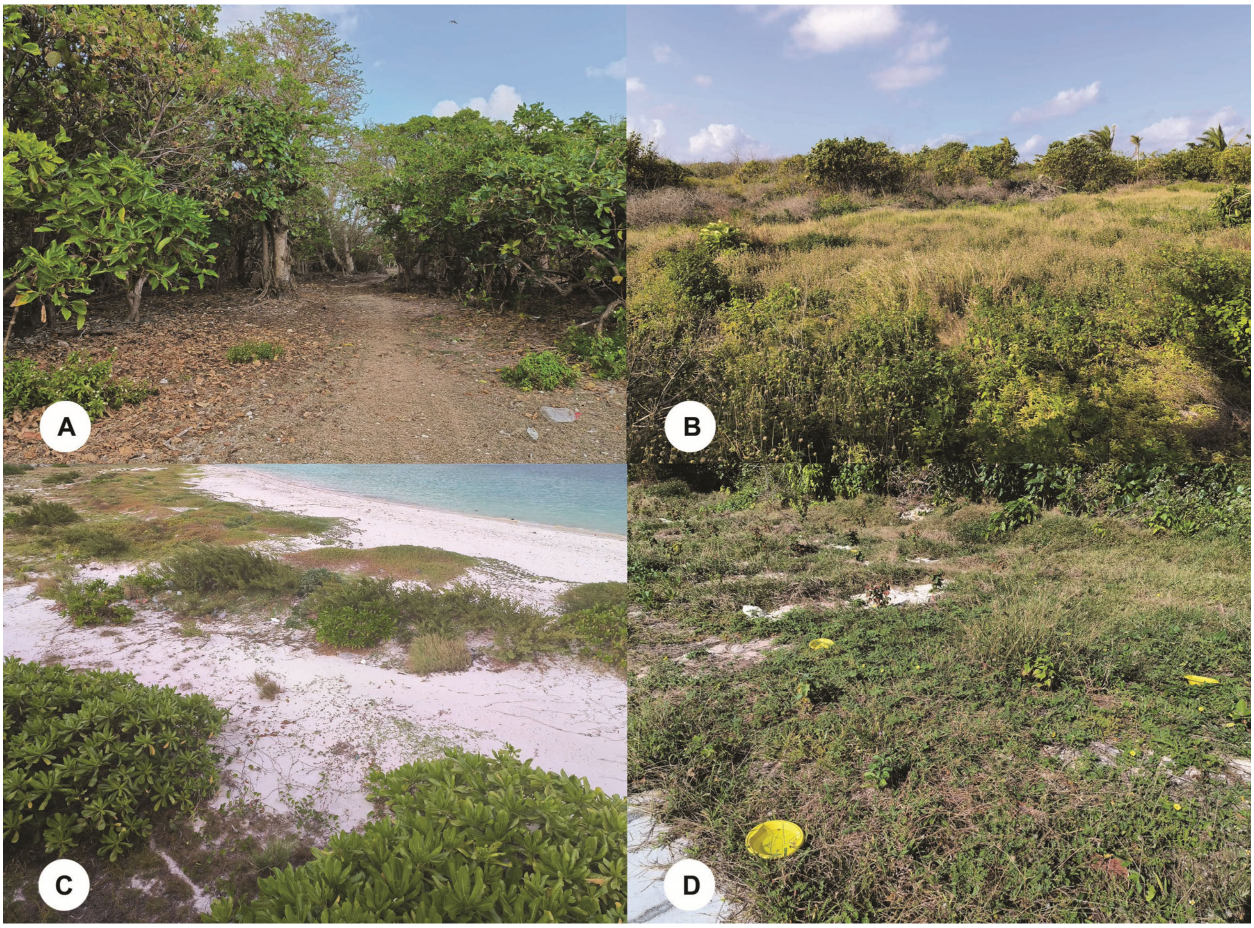

Figure S1. Different habitats of the Xisha Islands. (**A**) Forest. (**B**) Mixed forest, shrub and herbaceous community. (**C**) Costal shrub and herbaceous community. (**D**) Herbaceous community.

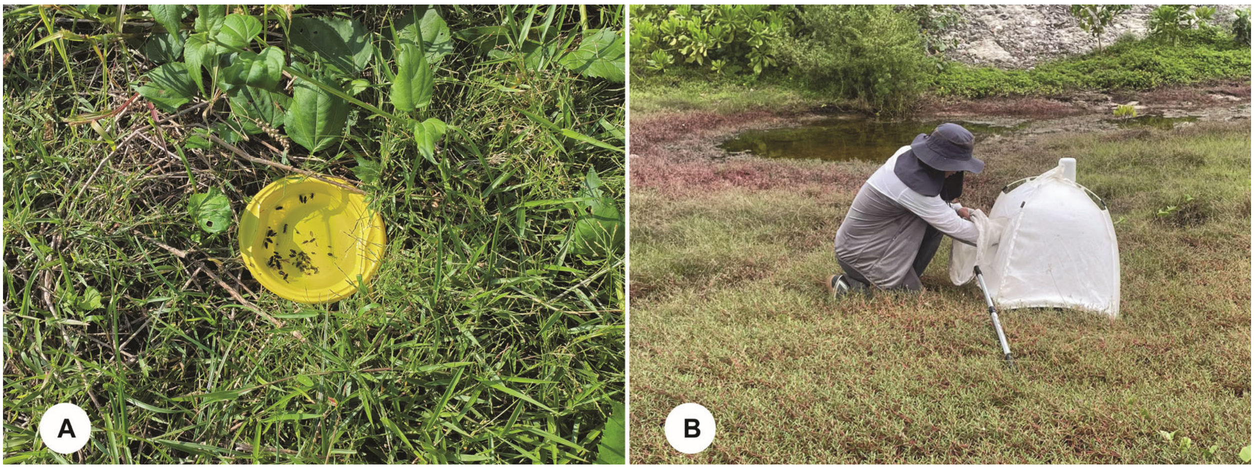

Figure S2. (**A**) Yellow pan trap. (**B**) Bottom-sealed emergence tent used with a sweep net and collecting tube.

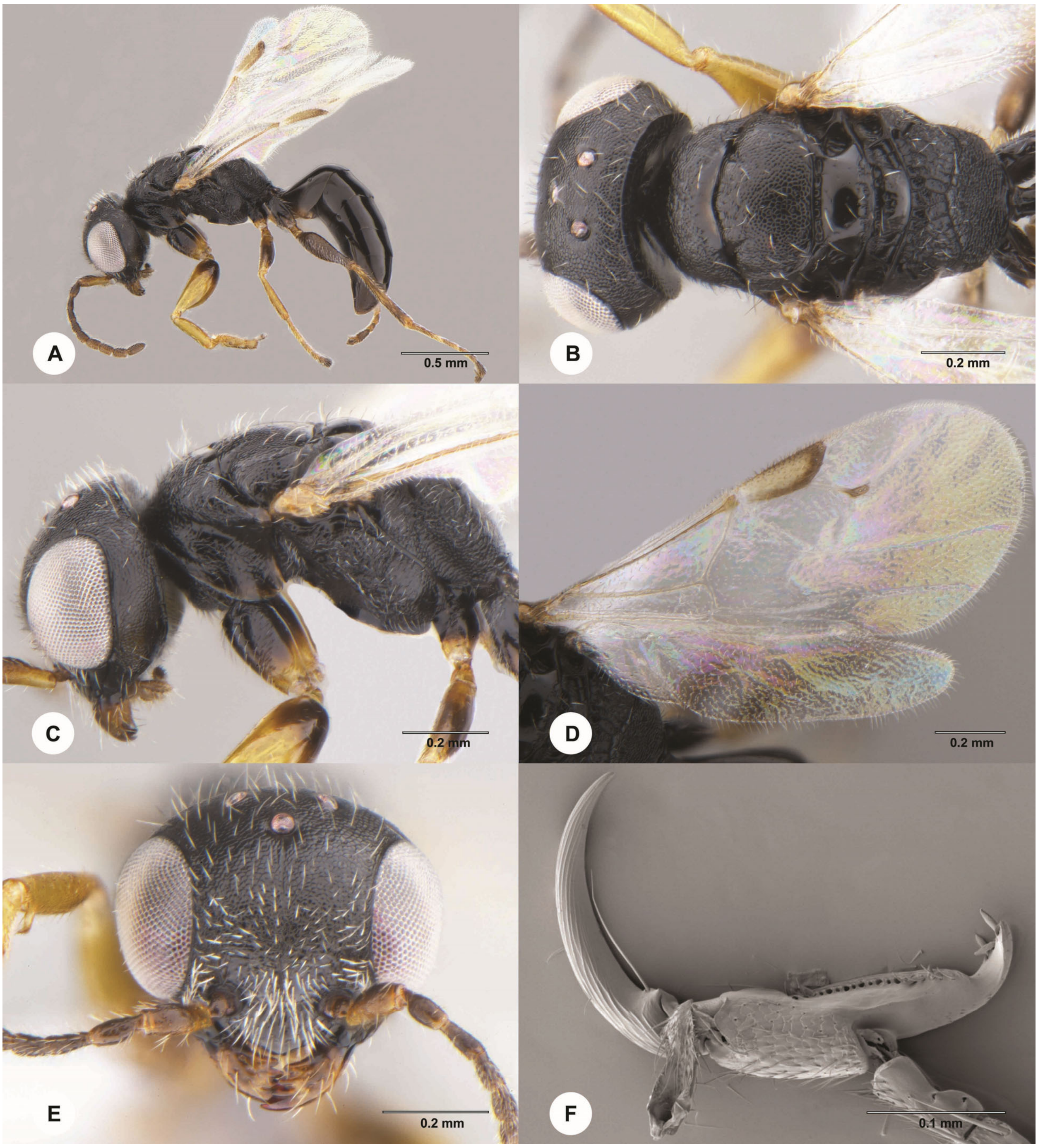

Figure S3. *Anteon malaysianum* Olmi, 1987, female (SCBG_E0009679). (**A**) Habitus, lateral view. (**B**) Head and mesosoma, dorsal view. (**C**) Head and mesosoma, lateral view. (**D**) Wings. (**E**) Head, frontal view. (**F**) Chela.

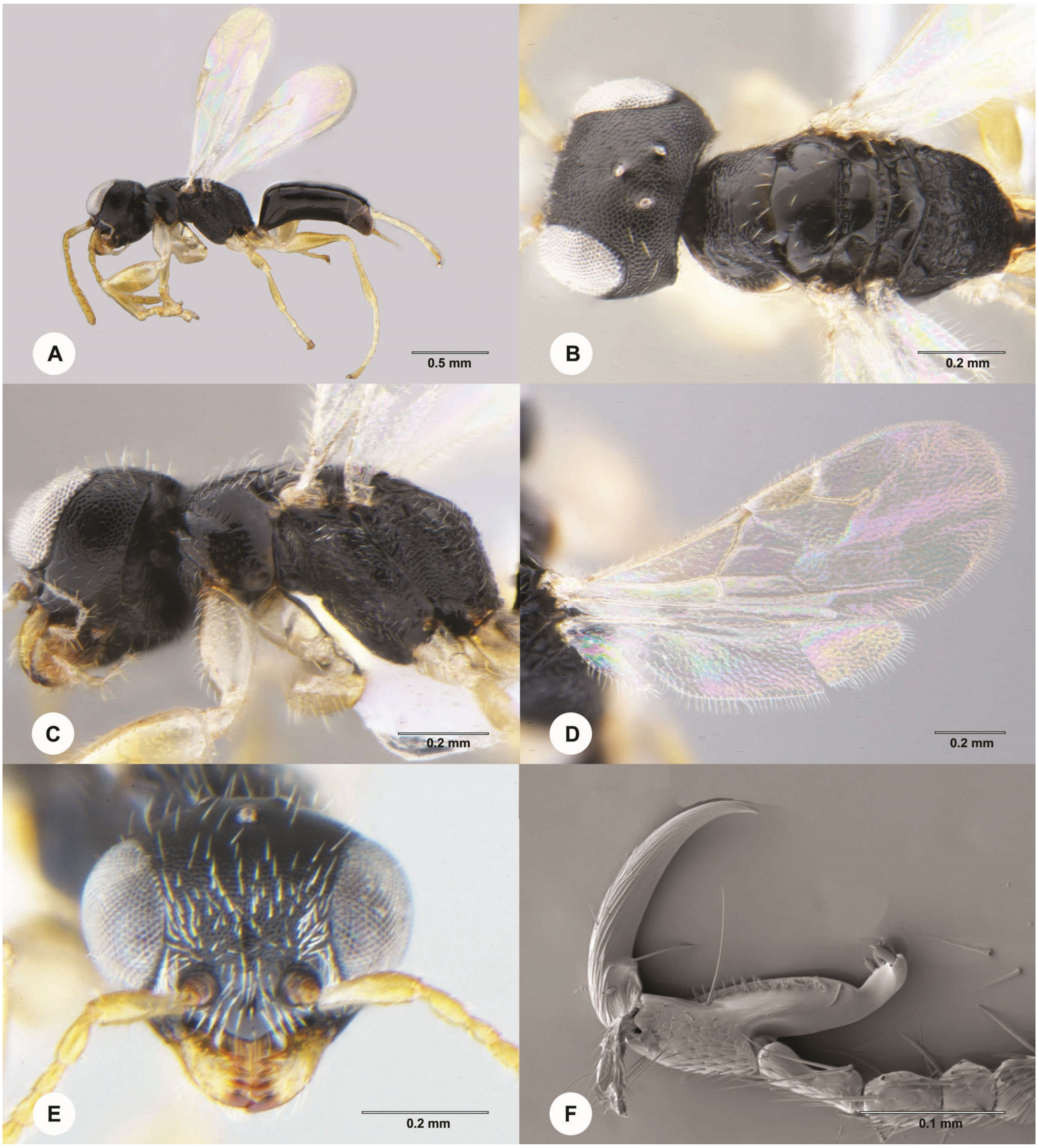

Figure S4. *Anteon yasumatsui* Olmi, 1984, female (SCBG_E0009680). (**A**) Habitus, lateral view. (**B**) Head and mesosoma, dorsal view. (**C**) Head and mesosoma, lateral view. (**D**) Wings. (**E**) Head, frontal view. (**F**) Chela.

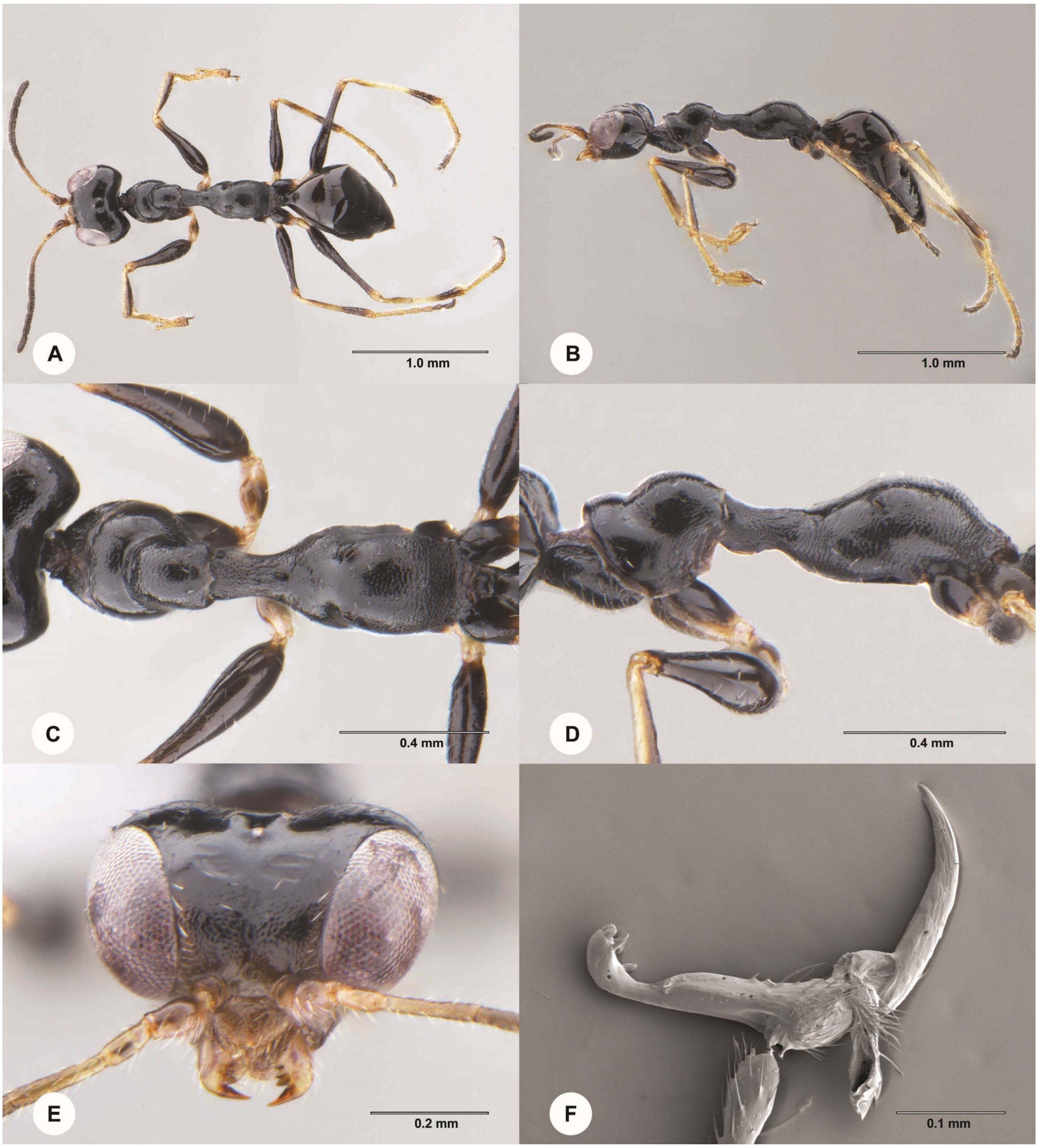

Figur S5. *Gonatopus nearcticus* (Fenton, 1905), female (SCBG_E0009681). (**A**) Habitus, dorsal view. (**B**) Habitus, lateral view. (**C**) Mesosoma, dorsal view. (**D**) Mesosoma, lateral view. (**E**) Head, frontal view. (**F**) Chela.

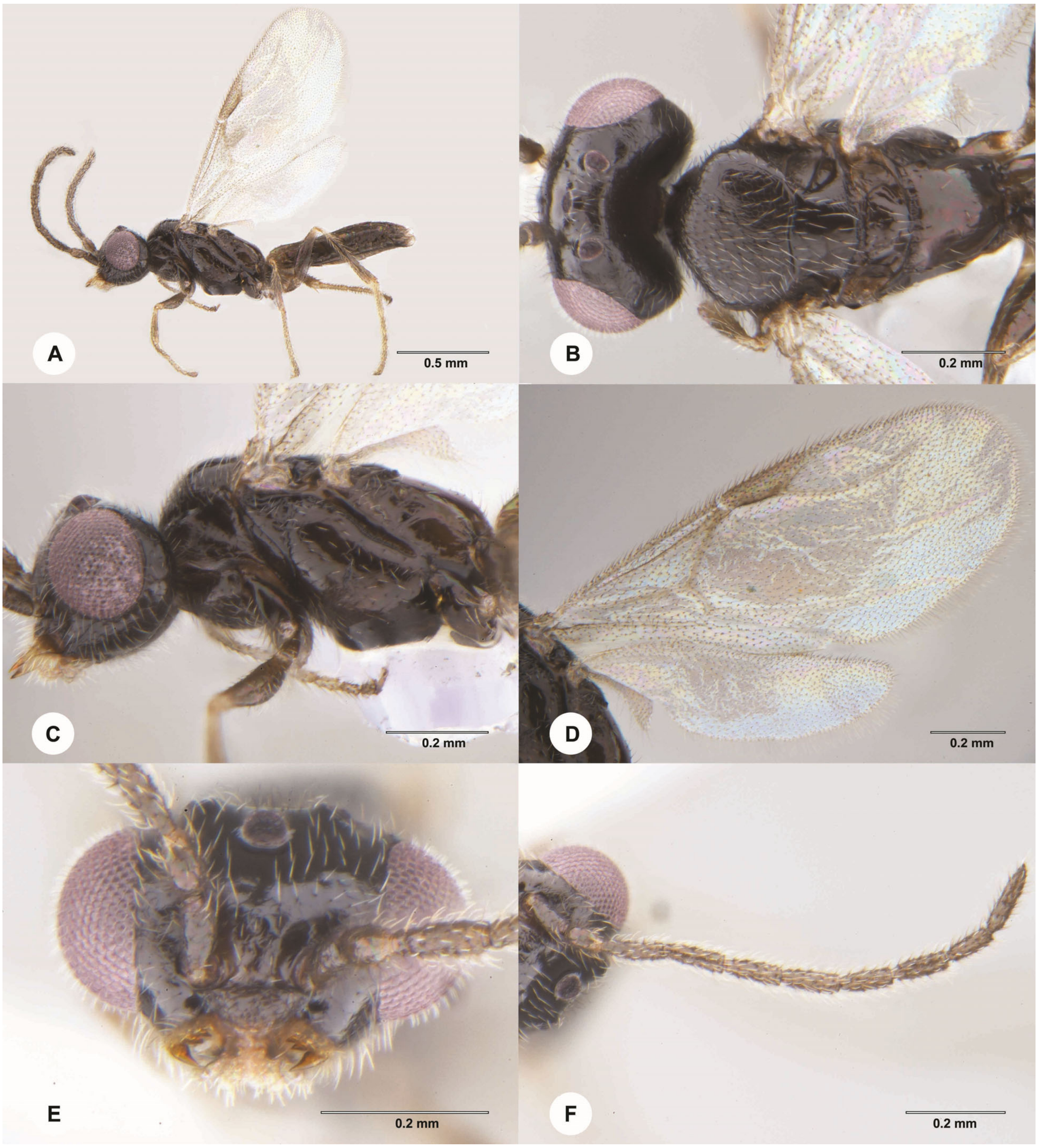

Figure S6. *Gonatopus nearcticus* (Fenton, 1905), male (SCBG_E0009682). (**A**) Habitus, larteral view. (**B**) Head and mesosoma, dorsal view. (**C**) Head and mesosoma, lateral view. (**D**) Wings. (**E**) Head, frontal view. (**F**) Antenna.

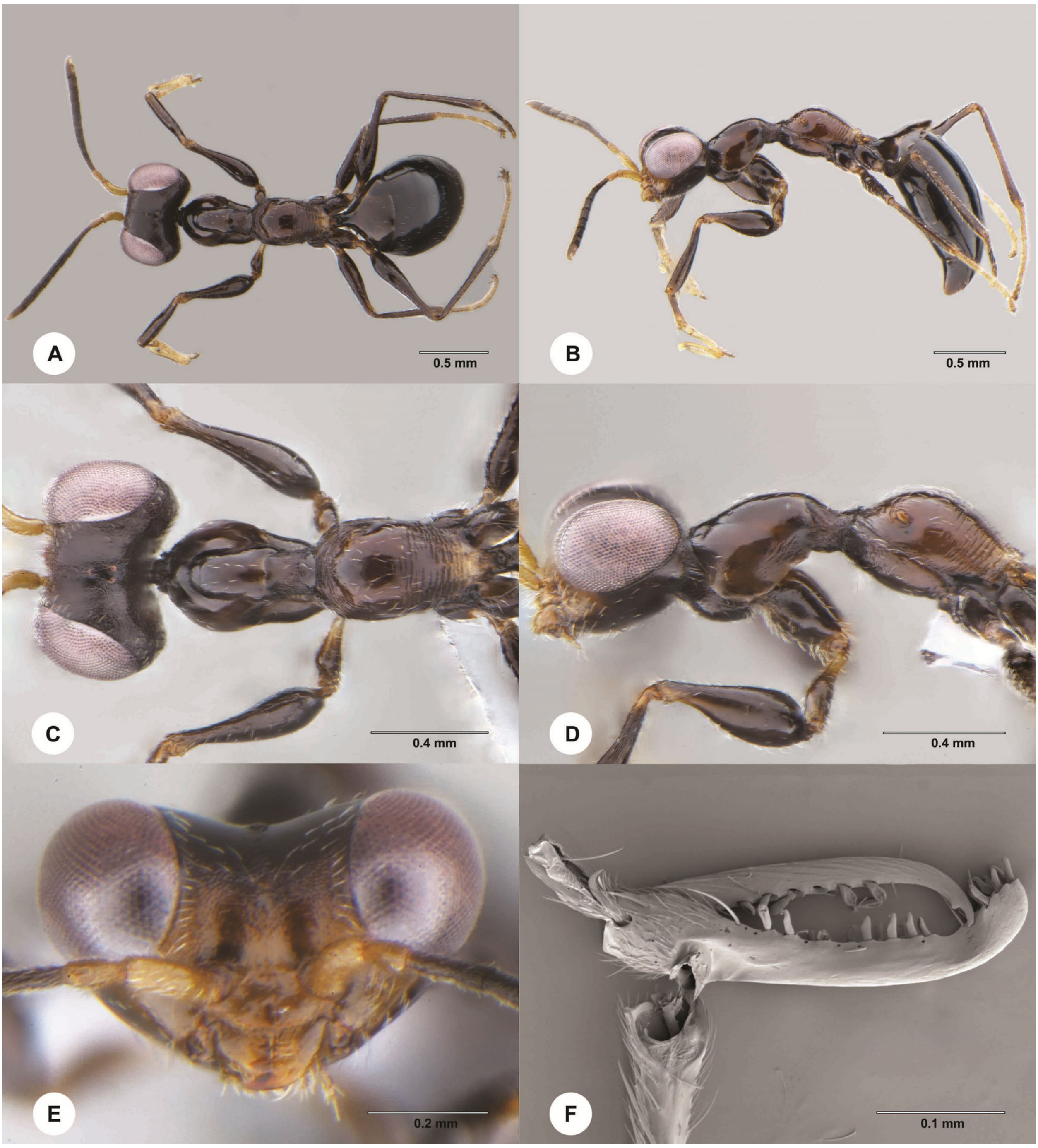

Figure S7. *Gonatopus validus* (Olmi, 1984), female (SCBG_E0009674). (**A**) Habitus, dorsal view. (**B**) Habitus, lateral view. (**C**) Head and mesosoma, dorsal view. (**D**) Head and mesosoma, lateral view. (**E**) Head, frontal view. (**F**) Chela.

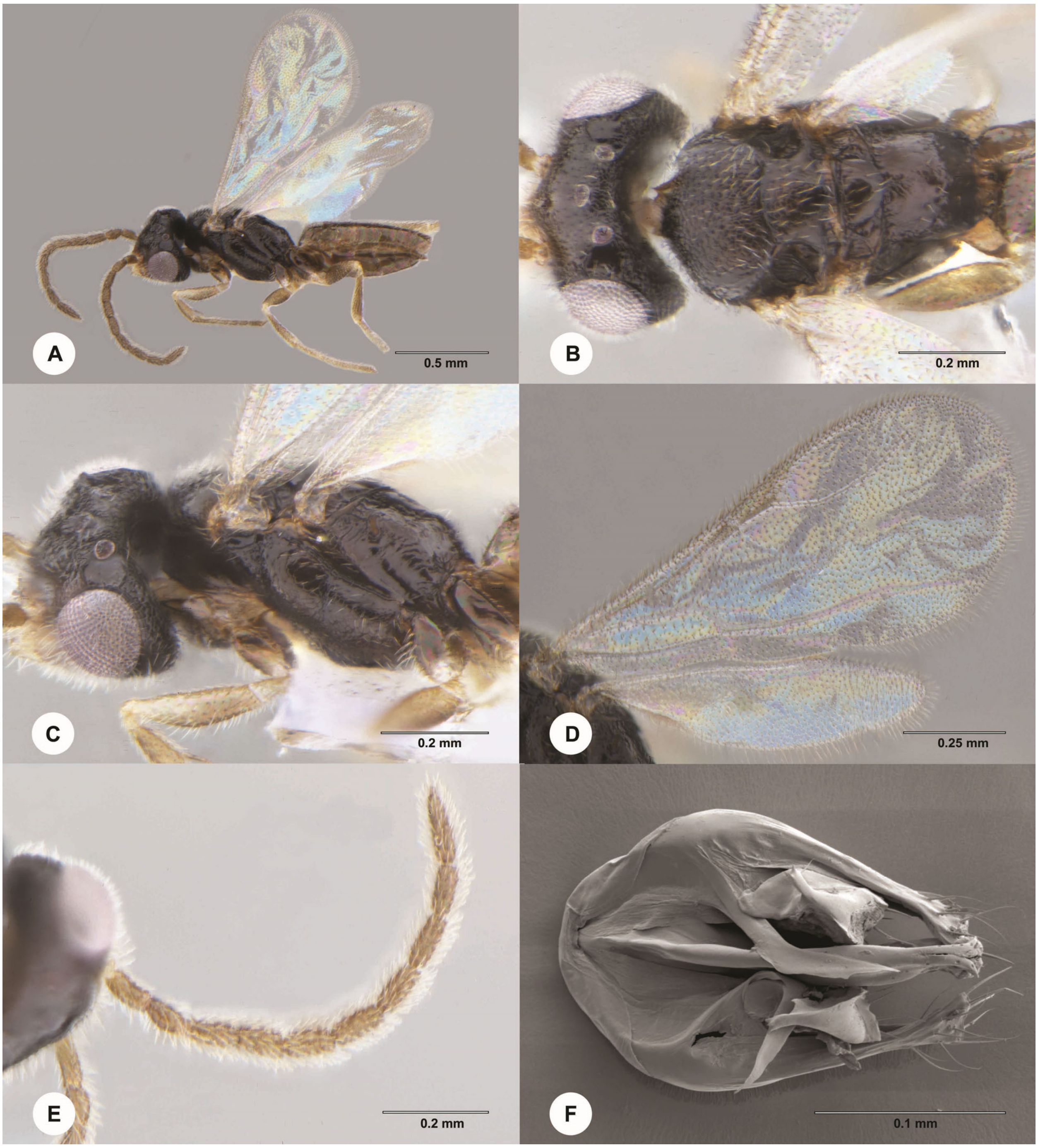

Figure S8. *Gonatopus validus* (Olmi, 1984), male (SCBG_E0009671). (**A**) Habitus, lateral view. (**B**) Head and mesosoma, dorsal view. (**C**) Head and mesosoma, lateral view. (**D**) Wings. (**E**) Antenna. (**F**) Genitalia, ventral view.

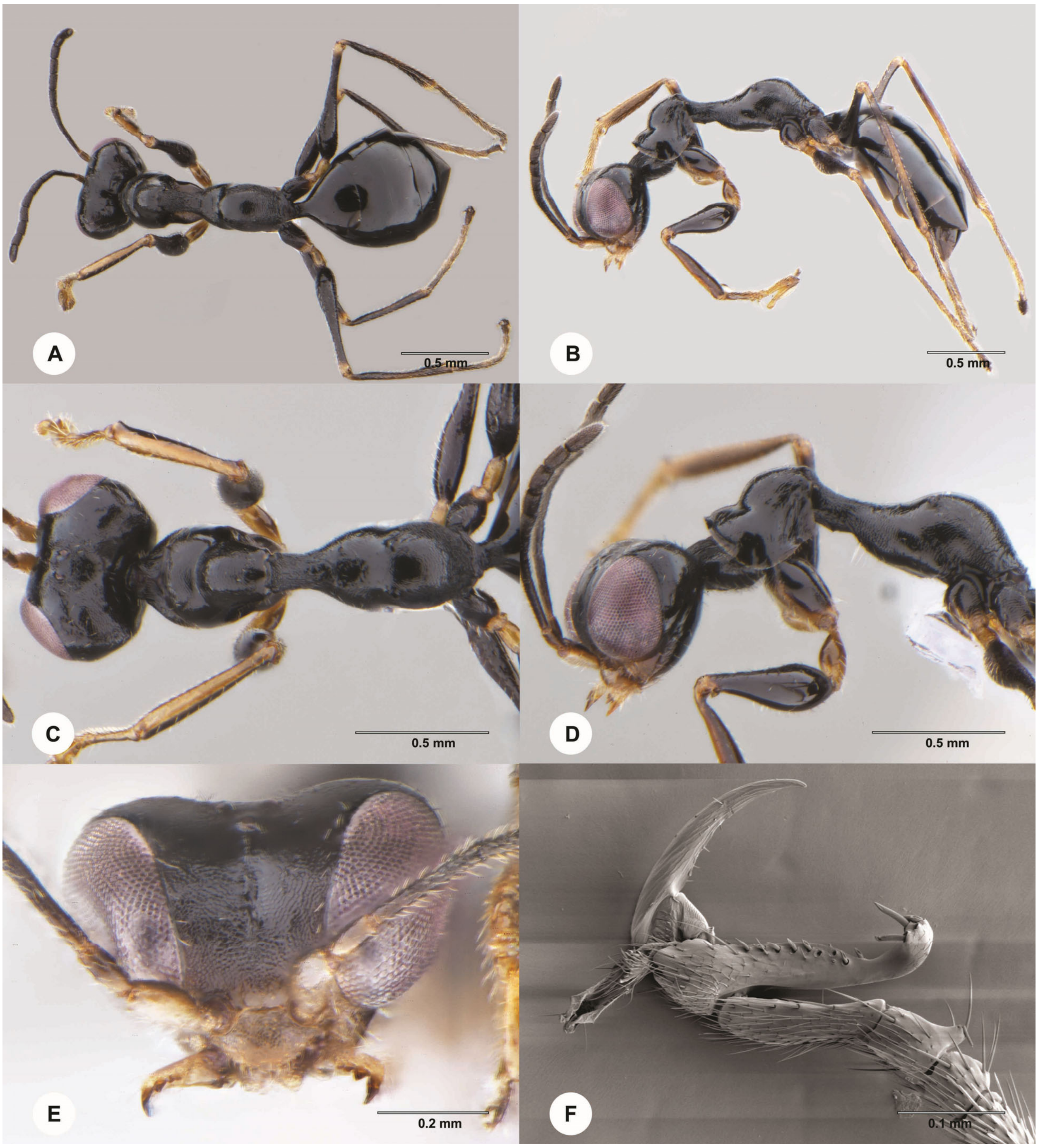

Figure S9. *Gonatopus yasumatsui* Olmi 1984, female (SCBG_E0009678). (**A**) Habitus, dorsal view. (**B**) Habitus, lateral view. (**C**) Head and mesosoma, dorsal view. (**D**) Head and mesosoma, lateral view. (**E**) Head, frontal view. (**F**) Chela.

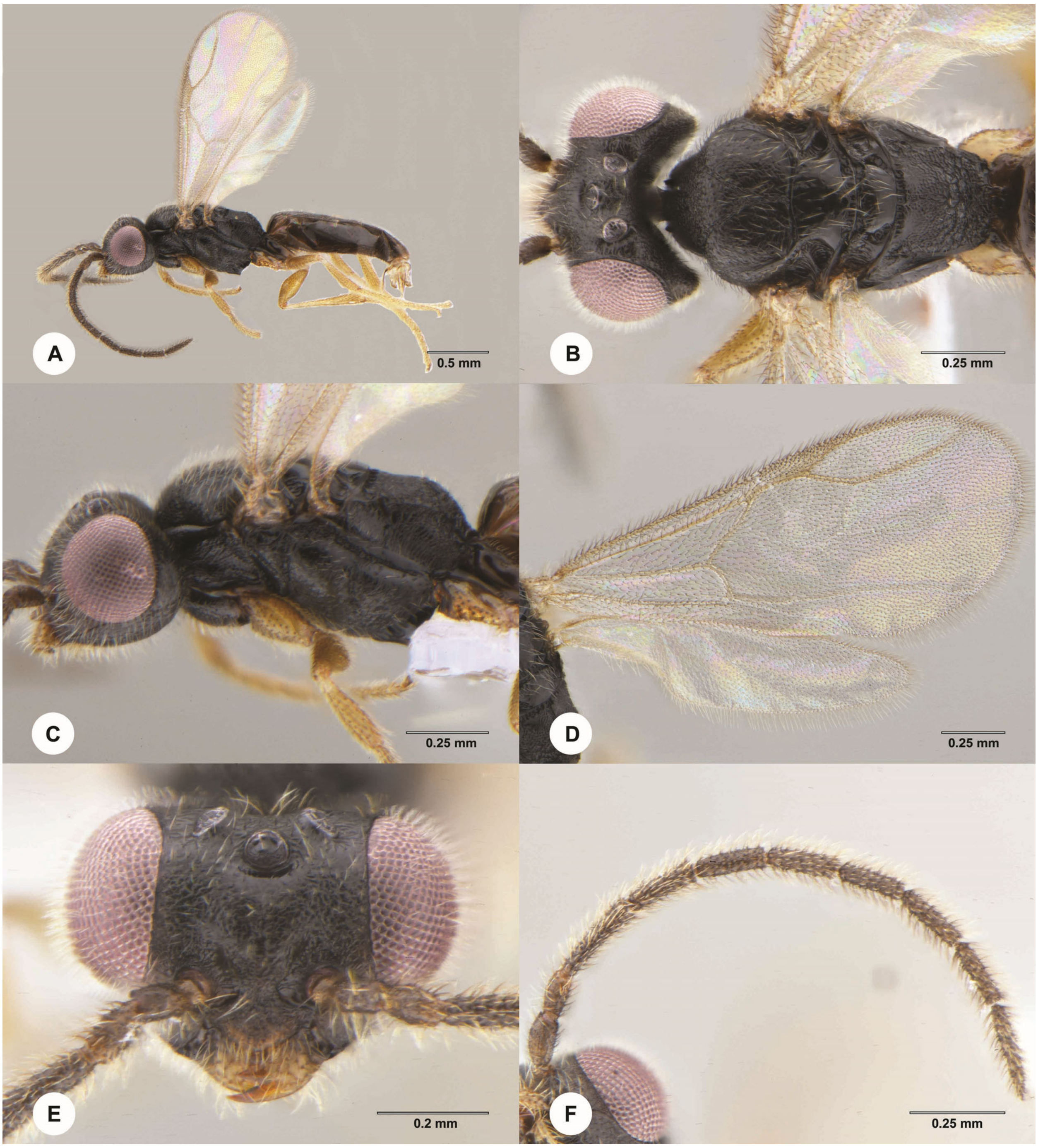

Figure S10. *Gonatopus* sp1, male (SCBG_E0009670). (**A**) Habitus, larteral view. (**B**) Head and mesosoma, dorsal view. (**C**) Head and mesosoma, lateral view. (**D**) Wings. (**E**) Head, frontal view. (**F**) Antenna.

Table S1. Geographic coordinates and area of sampled islets.

Islet	Location	Vegetated Area (km^2)
Yongxing Island	16°50′03″N 112°20′15″E	2.6
Jinqin Island	16°27′51″N 111°44′33″E	0.21
Chenhang Island	16°27′11″N 111°42′41″E	0.28
Pattle Island	16°32′14″N 111°36′25″E	0.31
Money Island	16°26′47″N 111°30′25″E	0.36
Lincoln Island	16°40′00″N 112°44′00″E	1.7
Robert Island	16°30′28″N 111°35′10″E	0.3
Zhaoshu Island	16°58′N 112°16′E	0.22
North Island	16°57′48″N 112°18′36″E	0.4
Antelope Reef	16°27′35″N 111°35′06″E	0.01

Table S2. Intraspecific pairwise distance of Dryinidae adults based on COI sequences (%)

Species	Distance (%)
Anteon chenhangense sp. n.	NA
Anteon yasumatsui	NA
Anteon malaysianum	0
Gonatopus nearcticus	0
Gonatopus yasumatsui	0
Gonatopus validus	0–1
Gonatopus sp1	NA

Table S3. Interspecific pairwise distance of Dryinidae adults based on COI sequences (%).

		1	2	3	4	5	6	7
1	*Anteon chenhangense* sp. n.		7.5	14	17.9	18.6	18.9–19.2	20.4
2	*Anteon yasumatsui*			12.3	18.4	18.6	18.5–18.6	18.8
3	*Anteon malaysianum*				19.7	18.6	19.5–19.7	20.6
4	*Gonatopus nearcticus*					7.6	13.7	14.8
5	*Gonatopus yasumatsui*						13.5	15.3
6	*Gonatopus validus*							13.5–13.6
7	*Gonatopus* sp1							

Table S4. Network metrics of the parasitoid-host network on the Xisha Islands.

Wasp species number	Host species number	Interaction number	Connectance	Specialization *H'2*	Nestedness
9	9	67	0.14	0.98	28.47
